# Long-Term PET-Nanoplastic Exposure Alters DNA Damage Response Capacity in BEAS-2B Human Bronchial Epithelial Cells

**DOI:** 10.3390/ijms27115031

**Published:** 2026-06-02

**Authors:** Michelle Morataya-Reyes, Aliro Villacorta, Raquel Egea, Joan Martín-Pérez, Javier Gutiérrez-García, Susana Pastor, Ricard Marcos, Alba Hernández

**Affiliations:** 1 Group of Mutagenesis, Department of Genetics and Microbiology, Faculty of Biosciences, Universitat Autònoma de Barcelona, 08193 Cerdanyola del Vallès, Spain; herlem.morataya@uab.cat (M.M.-R.); avillaco@unap.cl (A.V.); raquel.egea@uab.cat (R.E.); juan.martin.perez@uab.cat (J.M.-P.); javier.gutierrez.garcia@uab.cat (J.G.-G.); susana.pastor@uab.cat (S.P.); 2 Facultad de Recursos Naturales Renovables, Universidad Arturo Prat, Iquique 1100000, Chile

**Keywords:** nanoplastics, PET, long-term exposure, DNA damage response, genotoxicity

## Abstract

Chronic inhalation exposure to nanoplastics, specifically polyethylene terephthalate (PET) nanoplastics (PET-NPLs) is an emerging health concern, yet the long-term consequences for genomic stability and DNA damage response (DDR) capacity in bronchial epithelial cells remain poorly characterized. For this study, human bronchial epithelial BEAS-2B cells were continuously exposed to PET-NPLs for over 20 weeks, after which elevated basal DNA genotoxic damage was observed, as assessed by the alkaline comet assay. In addition, a broad transcriptional suppression of the DDR, with 27 of 84 profiled genes involved in DDR showing reduced expression relative to passage-matched control was observed. The suppressed genes span ATM/ATR checkpoint signaling, homologous recombination (HR), base excision repair (BER), nucleotide excision repair (NER), and apoptotic pathways. To determine whether chronic PET-NPL exposure altered susceptibility to acute genotoxic challenge in a damage-type-specific manner, cells were treated with methyl methanesulfonate (MMS), ultraviolet-C (UV-C) radiation, or bleomycin. While MMS and UV-C induced comparable levels of DNA damage in control and PET-exposed cells, bleomycin produced significantly greater damage in PET-exposed cells, indicating selective sensitization to doble-strand breaks (DSB)-type and oxidative genotoxic insults. Transcriptional profiling during bleomycin challenge identified 18 DDR genes with relatively higher expression in PET-exposed cells compared to passage-matched controls, encompassing HR, BER, ATM/ATR signaling, the Fanconi anemia pathway, and apoptosis. Furthermore, PET-exposed cells retained significantly higher residual DNA damage after 3 h of bleomycin challenge, indicating a persistent early repair deficit. Together, these findings suggest that chronic PET-NPL exposure specifically compromises the bronchial epithelial DDR, with potential implications for long-term genomic stability in respiratory epithelia subjected to nanoplastic inhalation.

## 1. Introduction

The ubiquitous nature of plastic pollution has shifted from an exclusively environmental concern to a critical human health issue. The use of plastic materials in daily life, and the resulting mismanagement of their waste, have led to the widespread release and dispersion of micro- and nanoplastics (MNPLs), particles ranging between 1–1000 µm (MPLs) and between 1 and 1000 nm (NPLs), respectively, resulting from the breakdown of big plastic fragments [1]. These particles can disperse in air, water, and even human food chains across ecosystems, making them a major emerging contaminant [2,3]. Polyethylene terephthalate (PET), one of the most widely produced synthetic polymers globally and the primary material in single-use bottles, food packaging, and synthetic textiles, is a dominant source of this kind of environmental contamination [4]. Inhalation represents a primary route of exposure, placing the bronchial epithelium at the frontline of potential NPL-induced toxicity [5,6]. Specifically, nanoplastics under 100 nm can translocate epithelial barriers reaching the bloodstream and accumulating in internal organs, including lungs [7,8].

While acute toxicity studies have provided initial insights, they often fail to capture the subtle, cumulative effects of chronic, low-dose exposure to NPLs that characterize real-world human scenarios [9,10]. The principal mechanism underlying genotoxicity of these particles is the generation of reactive oxygen species (ROS), which drives the formation of oxidized DNA bases, single-strand breaks and DNA double-strand breaks (DSBs) under conditions of high damage burden or replication fork collapse [11,12]. PET nanoplastics have been shown to induce ROS production and oxidative DNA damage in bronchial epithelial cell models at various concentrations [13,14]. However, most of the published genotoxicity studies have focused on acute or short-term exposure scenarios, leaving the consequences of chronic, repeated subtoxic exposure, which may produce the most biologically significant long-term alterations in cellular DNA damage response (DDR) capacity.

The DDR is a signaling network essential for maintaining genomic integrity and any impairment in its kinetics can lead to the accumulation of mutations and eventual neoplastic transformation [15]. Emerging evidence suggests that while nanoplastics may not always exhibit potent direct mutagenicity, they can interfere with cellular homeostasis and DDR [16,17]. Chronic exposure to subtoxic concentrations of nanoparticles may act as a “silent” stressor, potentially compromising the cell’s ability to respond to subsequent genotoxic insults, such as those originating from environmental pollutants or endogenous metabolic processes [18,19]. Rather than simply overwhelming repair capacity, persistent low-level genotoxic stress may progressively alter the steady-state expression of DDR effector genes, modify the balance between pro-repair and pro-apoptotic signaling, and, critically, change how cells respond to subsequent acute genotoxic insults [20]. These alterations are relevant to carcinogenesis risk, since chronic dysregulation of checkpoint and repair pathways is a recognized hallmark of early malignant transformation [21]. Despite this, the effect of chronic nanoplastic exposure on the DDR and on the capacity of chronically exposed cells to respond to well-defined acute genotoxic challenges has not been systematically characterized.

For the present study, the human bronchial epithelial cell line (BEAS-2B) was used as a respiratory model to evaluate the effects of long-term (20-week) exposure to environmentally representative (real-life) PET nanoplastics (50 µg/mL). This prolonged exposure model allowed for systematic assessment of the cumulative effects of chronic PET-NPLs exposure on basal genomic stability and the response to defined genotoxic challenges of distinct mechanistic classes. A critical and underexplored dimension of NPLs genotoxicity is whether chronic exposure produces changes in damage-type-specific susceptibility, meaning if the cellular capacity to respond to different classes of DNA lesions is differentially affected. Alkylating agents such as methyl methanesulfonate (MMS) generate base adducts primarily processed by base excision repair (BER); ultraviolet-C (UV-C) radiation produces bulky pyrimidine dimers repaired by nucleotide excision repair (NER); while bleomycin, a radiomimetic glycopeptide antibiotic, generates complex oxidative lesions and DSBs that activate the ATM/ATR checkpoint axis and engage homologous recombination (HR). To evaluate the molecular basis of this damage-type-specific response, the transcriptional response in both PET-exposed and passage-matched control cells was profiled using the RT^2^ Profiler™ PCR Array Human DNA Damage Signaling Pathway including 84 DDR genes across ATM/ATR checkpoint signaling, homologous recombination, NER, BER, Fanconi Anemia pathway, and apoptosis. A complementary alkaline comet assay was used to measure genotoxic damage at the basal level and after the induction of the different types of damage. By employing this approach, a comprehensive analysis is presented of the effect of long-term PET-NPLs exposure on genomic stability and DDR in BEAS-2B cell model.

## 2. Results and Discussion

### 2.1. Characterization of PET-NPLs and Internalization in BEAS-2B Cells

Before evaluating the biological effects of chronic PET-NPL exposure on the DNA damage response (DDR), the particles’ physicochemical properties were characterized to confirm their suitability as an environmentally real-life representative nanoplastic model. Size distribution was assessed by dynamic light scattering (DLS) and nanoparticle tracking analysis (NTA), while morphology was evaluated by transmission electron microscopy (TEM). Cellular internalization after 20 weeks of continuous exposure was confirmed by confocal microscopy.

**Figure 1:**
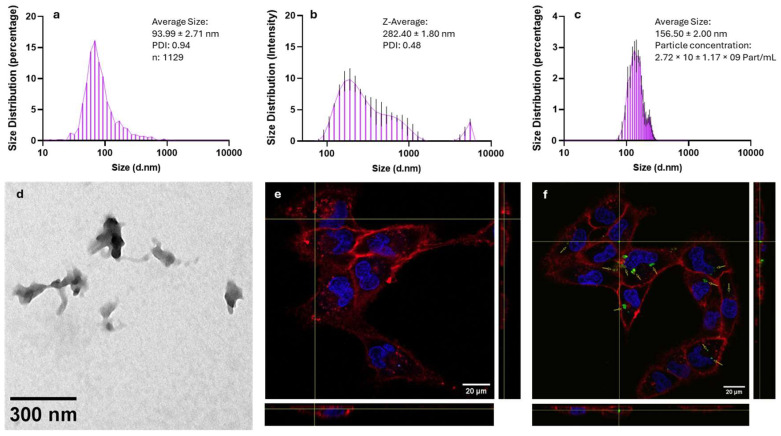
Characterization of PET-NPLs and internalization in BEAS-2B cells after 20 weeks. (**a**) Size distribution of PET-NPLs by Martin diameter (TEM) and (**b**) Dynamic light scattering (DLS) size distribution by intensity, (**c**) Nano tracking analysis (NTA), showing particle concentration and size distribution. (**d**) TEM images of the PET showing the irregular shape and heterogeneous size of the particles. (**f**) Confocal microscopy evidencing the internalization of PET-NPLs in BEAS-2B cells after 20 weeks of continuous exposure to the particles, compared to non-exposed passage controls (**e**).

TEM imaging analysis showed a size distribution of 93.99 ± 2.71 nm with a polydispersity index of 0.94, confirming the heterogenous nature of the samples make it more realistic to what is expected to be found in nature. Additionally, the irregular, non-spherical morphology of the PET-NPLs was observed (Figure 1a,d). The heterogeneous morphology of the particles observed here is characteristic of secondary nanoplastics considering the top-down fragmentation approach rather than controlled synthesis and has been associated with differential cellular interactions compared to spherical model particles, including altered endocytic uptake dynamics and intracellular trafficking [16,22].

DLS and NTA data provided complementary size distribution related to the hydrodynamic behavior of PET-NPLs (Figure 1b,c), showing an average size bigger than the dry state (as expected), from 282.40 ± 1.80 nm on DLS to 156.50 ± 2.00 nm on NTA, possibly due to the resolution differences between the techniques. The PDI on DLS was 0.48, confirming the heterogeneity observed on the TEM imaging and the particle concentration from NTA was 2.72 × 10^10^ ± 1.17 × 10^9^ particles/mL. These findings along with a ζ-potential of −29.1 reveal that the particle population not only falls into the nano range but is also sensitive to the presence of larger agglomerates or aggregates in the heterogeneous preparation, consistent with the expected characteristics of secondary nanoplastics generated from mechanical fragmentation of bulk polymer [23]. This heterogeneity is considered environmentally relevant, as real-world nanoplastic contamination does not consist of monodisperse populations.

Following 20 weeks of continuous exposure to 50 µg/mL, internalization of PET-NPLs in BEAS-2B cells was confirmed by confocal microscopy. Non-exposed passage-matched control cells showed no detectable fluorescent signal in the cytoplasmic compartment (Figure 1e). In contrast, PET-NPL-exposed cells exhibited a clear intracellular fluorescent signal corresponding to the particles, distributed predominantly in the perinuclear region (Figure 1f). This intracellular accumulation pattern is consistent with previous reports of nanoplastic uptake via endocytosis, followed by trafficking to late endosomes and lysosomes [24]. The sustained internalization of PET-NPLs over the 20-week exposure period is a prerequisite for the chronic intracellular stress that would be expected to modulate DNA damage response pathways, as discussed below. Critically, the absence of fluorescent signal in the nucleus indicates that the particles did not directly access genomic DNA, suggesting that any observed genotoxic or transcriptional effects could be mediated indirectly, through cytoplasmic mechanisms such as oxidative stress, lysosomal damage, or interference with cytoskeletal and signaling components, rather than through direct particle–DNA interaction [18,25].

### 2.2. Effects of Long-Term Exposure to PET-NPLs in Basal Genomic Integrity and DDR Gene Expression

**Figure 2:**
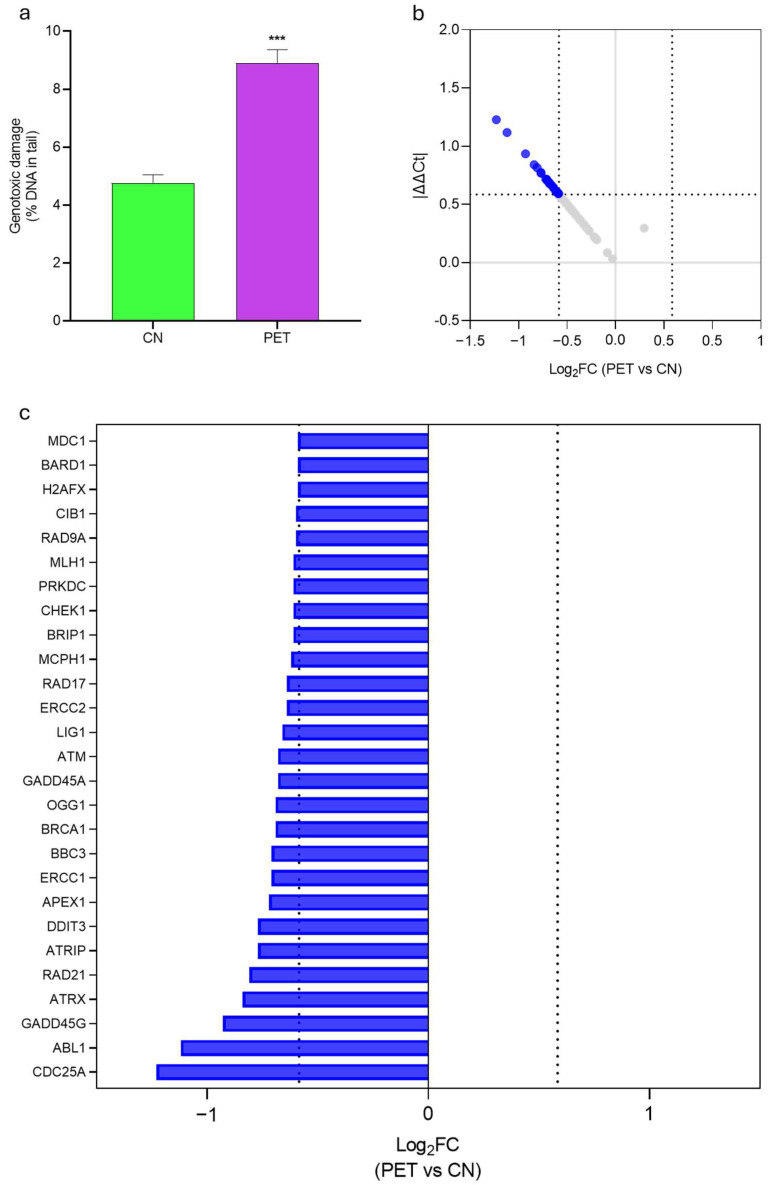
Basal genomic instability and attenuated DDR gene expression after chronic PET-NPL exposure. (**a**) Alkaline comet assay showing elevated basal DNA damage in BEAS-2B cells after 20 weeks of continuous exposure to 50 µg/mL PET-NPLs (purple) compared to passage-matched control cells (green). (**b**) Scatterplot of DDR expression showing the relationship between Log_2_FC (x-axis) and |ΔΔCt| magnitude (y-axis) of DDR gene expression (84 genes) in PET-exposed versus control cells, showing broad downregulation at baseline (27 genes with Log_2_FC < −0.585). Downregulated genes in PET-exposed cells are shown in blue and genes with no detectable change are in gray. (**c**) Bar plot of 27 downregulated genes, organized by DDR pathway classification (ATM/ATR checkpoint signaling, homologous recombination, nucleotide excision repair, base excision repair, DSB sensing, mismatch repair, and apoptotic signaling). Data in (**a**) represent mean ± SEM; *n* = 3 biological replicates; Mann–Whitney U test; *** *p* ≤ 0.001.

DNA damage levels and DDR gene expression were evaluated to determine the basal phenotype of BEAS-2B cells after 20 weeks of continuous exposure to 50 µg/mL of PET-NPLs (PET) and that of passage-matched control cells (CN). Alkaline comet assay revealed that PET-exposed cells carried significantly higher levels of endogenous DNA damage than controls, showing approximately 1.9-fold higher genotoxic damage (Figure 2a; 8.91 ± 0.46% DNA in tail for PET vs. 4.77 ± 0.28% for controls, mean ± SEM, *p* < 0.001) and establishing that chronic, low-dose PET-NPLs exposure at subtoxic concentrations produces cumulative genomic stress without triggering acute cytotoxic responses. This is consistent with previous reports showing that PET and other plastic particles produce reactive oxygen species (ROS) in bronchial epithelial models, which drive oxidative DNA lesions and single-strand breaks [13,14,26,27].

**Figure 3:**
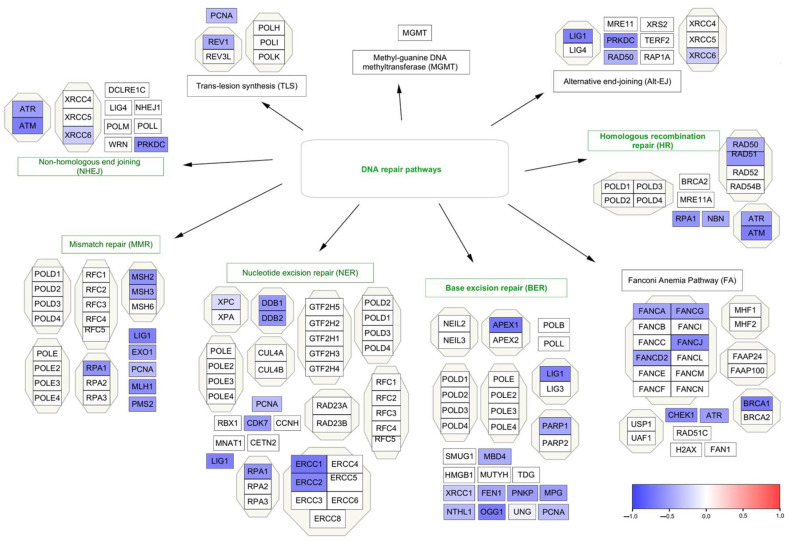
Mapping of baseline DDR gene expression changes in PET-NPL-exposed BEAS-2B cells onto the DNA Repair Pathways Full Network (WikiPathways WP4946). Gene expression was compared between passage-matched control (CN) and PET-NPL-exposed (PET) BEAS-2B cells after 20 weeks of continuous exposure in the absence of genotoxic challenge, using the RT^2^ Profiler™ PCR Array Human DNA Damage Signaling Pathway. The network visualization illustrates that the transcriptional suppression associated with chronic PET-NPL exposure is not confined to a single repair pathway but spans multiple DDR components simultaneously, including ATM/ATR checkpoint signaling, homologous recombination, base excision repair, nucleotide excision repair, and non-homologous end joining. Gene nodes within the network are colored according to the Log_2_FC of PET-exposed relative to control cells: blue indicates downregulation in PET-exposed cells, with color intensity proportional to the magnitude of suppression (Log_2_FC ≤ −0.585); red indicates upregulation (Log_2_FC ≥ +0.585); nodes with no detectable change (|Log_2_FC| < 0.585) are shown in white (*n* = 3 biological replicates).

At the transcriptional level, profiling of 84 DDR genes revealed a broad suppression of DDR gene expression in PET-exposed cells compared to passage controls (Figure 2b,c). A total of 27 of the 84 profiled genes were downregulated in PET-exposed cells, with no genes exceeding the 1.5-fold upregulation threshold (|Log_2_FC| > 0.585). The most strongly suppressed genes were *CDC25A* (Log_2_FC = −1.23, FC = 0.43) and *ABL1* (Log_2_FC = −1.12, FC = 0.46), two key coordinators of cell cycle checkpoint activation downstream of DDR signaling [28,29], and their chronic downregulation may contribute to an attenuated checkpoint response upon acute challenge with genotoxic agents. The 27 downregulated genes collectively spanned ATM/ATR checkpoint signaling (*ATM*, *ATRIP*, *CHEK1*, *RAD17*, *BARD1*, *RAD9A*), homologous recombination (*HR*; *BRCA1*, *BRIP1*, *RAD21*, *ATRX*), nucleotide excision repair (*NER*; *ERCC1*, *ERCC2*), base excision repair (*BER*; *APEX1*, *OGG1*, *LIG1*), DSB sensing (*H2AFX*, *MDC1*, *PRKDC*), mismatch repair (*MLH1*), and apoptotic signaling (*BBC3*, *DDIT3*, *CIB1*) (Figure 2c, full gene list with fold changes is provided in Supplementary Table S1). The profile demonstrates that, at baseline, chronic PET-NPL exposure is associated not only with elevated endogenous DNA damage but also with an attenuated DDR transcriptome across multiple repair and checkpoint pathways (Figure 3).

The combination of elevated basal genomic damage and systematic downregulation of DDR gene expression at baseline shows a dual vulnerability state, consistent with the concept of a “silent” stressor, a persistent, subtoxic perturbation that gradually diminishes the cell’s capacity to maintain genomic integrity without triggering overt cell death or senescence signals [18,19]. The finding of simultaneous downregulation across diverse DDR pathways, rather than pathway-specific compensation, suggests that chronic exposure not only creates a source of ongoing DNA damage but simultaneously reprograms the transcriptional machinery responsible for responding to that damage. This transcriptional reprogramming likely reflects sustained oxidative stress-induced alterations in chromatin remodeling, epigenetic landscapes, and the activity of key transcription factors regulating DDR genes. Sustained oxidative stress is known to alter chromatin remodeling, epigenetic landscapes, and the activity of transcription factors regulating DDR genes, including those dependent on p53 and ATM/ATR signaling [30,31,32]. Notably, the baseline downregulation included multiple p53-target genes (e.g., *GADD45A*, *GADD45G*, *BBC3*, *BAX*) and ATM/ATR-regulated genes (*ATM*, *ATR*, *CHK1*, *CHK2*, *ATRIP*), suggesting that chronic PET exposure may suppress p53 activation or availability, or alternatively, may push the cell toward a chronic low-level p53 state that is insufficient to mount effective compensatory responses (Figure S1). The concurrent downregulation across both the repair genes and the apoptotic genes of DDR is unusual and could suggest a state of metabolic or signaling exhaustion rather than selective pathway inhibition, since under normal circumstances, cells experiencing DNA damage would activate either repair or apoptotic pathways in a dose-dependent manner.

### 2.3. Damage-Type-Specific Sensitivity of PET-Exposed BEAS-2B

To determine whether chronic PET-NPL exposure altered cellular susceptibility to exogenous genotoxic challenge, and whether any such alteration was damage-type-specific, PET-exposed and passage-matched control cells were treated with three mechanistically distinct genotoxic agents: methyl methanesulfonate (MMS), an alkylating agent that generates base adducts primarily repaired by base excision repair (BER) [33]; ultraviolet-C (UVC) radiation, which induces bulky pyrimidine dimers processed by nucleotide excision repair (NER) [34]; and bleomycin, a radiomimetic agent that generates complex oxidative lesions and DNA double-strand breaks (DSBs) repaired by ATM/ATR-dependent pathways and homologous recombination [35].

**Figure 4:**
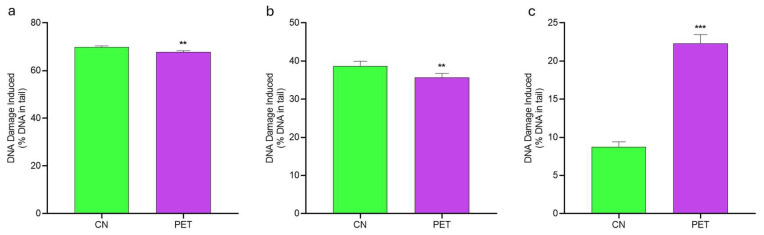
Selective sensitization of PET-exposed BEAS-2B cells to bleomycin-induced double-strand break damage. DNA damage induced by three mechanistically distinct genotoxic agents in passage-matched control cells (green) and PET-exposed cells (purple). (**a**) MMS treatment (alkylating agent, BER-dependent): statistically significant but small absolute difference in damage (~2.1%, *p* ≤ 0.01), with comparable high damage levels in both groups indicating functional BER capacity is preserved. (**b**) UV-C radiation (bulky adducts, NER-dependent): statistically significant but biologically similar damage levels (~3.1% difference, *p* = 0.004), indicating functional NER capacity is preserved. (**c**) Bleomycin treatment (radiomimetic agent, ATM/ATR-HR-dependent): substantially elevated damage in PET-exposed cells (~2.6-fold increase), revealing selective vulnerability to complex DSB-type lesions. Data represent mean ± SEM; *n* = 3 biological replicates; Mann–Whitney U test; ** *p* ≤ 0.01, *** *p* ≤ 0.001.

The results revealed a clear damage-type specificity. Following MMS treatment (1 µM, 30 min), the levels of induced DNA damage statistically differ between CN and PET-exposed cells (Figure 4a, PET: 67.79 ± 0.55% DNA in tail vs. CN: 69.93 ± 0.45%, mean ± SEM, *p* ≤ 0.01); however, the absolute difference is of only around 2.1% of DNA in the comet tail, indicating that the cellular capacity to respond to alkylation-induced lesions is functionally the same in both groups. Similarly, UV-C irradiation (10 mJ/cm^2^) produced statistically different levels of DNA strand breaks in both groups (Figure 4b; PET: 35.69 ± 1.08% DNA in tail vs. CN: 38.74 ± 1.20%, mean ± SEM, *p* = 0.004), with an absolute difference of around 3.1% of DNA in the tail. Despite these statistical significances, the damage levels are biologically comparable and indicate that BER- and NER-dependent damage sensitivity was not substantially affected by chronic PET exposure. In contrast, bleomycin treatment (25 µg/mL, 1 h) produced a significantly greater level of DNA damage in PET-exposed cells compared to controls (Figure 4c; PET: 22.34 ± 1.13% DNA in tail vs. CN: 8.72 ± 0.70%, mean ± SEM, *p* ≤ 0.001), approximately a 2.6-fold increase. This selective sensitization of PET-exposed cells to bleomycin-induced DSB-type damage, in the absence of generalized hypersensitivity to BER- or NER-dependent lesions, reveals a complex relationship between baseline DDR suppression and functional vulnerability that would not be apparent from genotoxicity studies employing a single damaging agent.

This damage-type specificity can be mechanistically understood through the differential complexity and pathway requirements of the three lesion types. Bleomycin induces complex lesions involving single-strand breaks, double-strand breaks, and oxidative modifications whose repair is dependent on the ATM/ATR checkpoint cascade and homologous recombination (HR) pathways, which are the pathways showing the most dramatic downregulation at baseline in PET-exposed cells (Figure 2b). The selective vulnerability to bleomycin-type lesions suggests that the baseline suppression of ATM/ATR-dependent checkpoint and HR genes could create a bottleneck specifically for complex DSB-type damage. In contrast, MMS-induced lesions are predominantly processed by base excision repair (BER), which, while downregulated at baseline, appears resilient to transcriptional suppression and may likely benefit from substrate-driven induction or sufficiently abundant in baseline pools to meet the acute challenge demand. Similarly, UV-C-induced pyrimidine dimers are handled by nucleotide excision repair (NER), though downregulated at baseline, did not show impaired capacity in the challenge experiment. This suggests that simple, linear repair pathways (BER, NER) are more resilient to the baseline transcriptional suppression observed after chronic exposure to PET-NPLs, possibly because they operate at high activity levels or are more effectively compensated by non-transcriptional mechanisms [36,37].

### 2.4. Transcriptional Response and DDR of PET-Exposed BEAS-2B After Bleomycin Challenge

**Figure 5:**
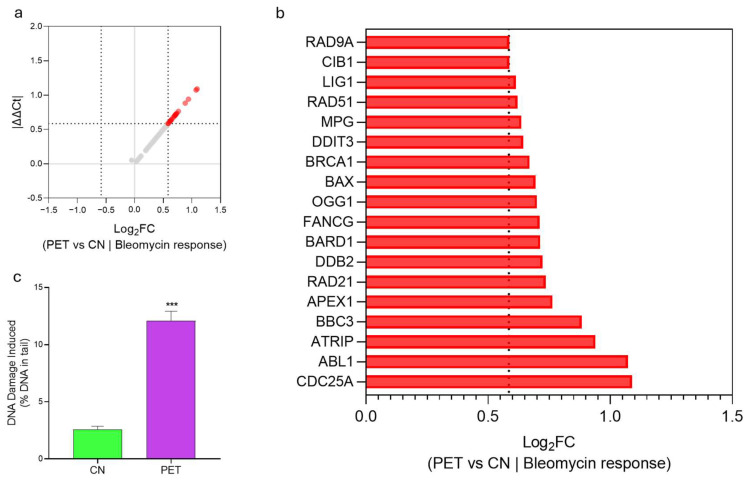
Transcriptional upregulation and impaired repair response following bleomycin challenge. Differential gene expression and repair capacity in PET-exposed vs. control cells following bleomycin-induced DSB damage. (**a**) Scatterplot of DDR expression showing the relationship between Log_2_FC (x-axis) and |ΔΔCt| magnitude (y-axis) of 18 genes with higher relative expression in bleomycin-treated PET-exposed cells compared to bleomycin-treated control cells (ΔΔCt analysis), with no genes showing lower relative expression. Upregulated genes are shown in blue, and genes with no detectable change are gray. (**b**) Bar plot of the 18 upregulated genes, organized by DDR pathway, showing fold-changes ranging from approximately 1.6- to 2.1-fold (Log_2_FC 0.7–1.1). (**c**) Residual DNA damage measured after 3 h of repair time, showing persistent genotoxic damage in PET-exposed cells despite the elevated transcriptional response observed in (**a**,**b**). Data represent mean ± SEM; *n* = 3 biological replicates; Mann–Whitney U test; *** *p* ≤ 0.001.

To gain mechanistic insight into the selective bleomycin sensitization observed in PET-exposed cells, the transcriptional response to bleomycin challenge was characterized using the ΔΔCt method, which quantifies the differential response of PET-exposed cells relative to control cells beyond the shared genotoxin-induced response. Cells were harvested immediately upon completion of bleomycin treatment, capturing the transcriptional state during acute DSB-type damage. This analysis identified 18 genes with significantly higher relative expression in PET-exposed cells following bleomycin challenge (Figure 5a,b; Table S1), with no genes showing significantly lower expression. The differentially expressed genes spanned a broad spectrum of DDR functions (Figure 5b): ATM/ATR checkpoint signaling (*ABL1*, *ATRIP*, *BARD1*, *RAD9A*), *HR* (*BRCA1*, *RAD51*, *RAD21*); BER (*APEX1*, *OGG1*, *MPG*, *LIG1*); NER (*DDB2*); Fanconi Anemia pathway (*FANCG*); and apoptotic/stress signaling (*BBC3*, *BAX*, *DDIT3*, *CIB1*); with fold-changes ranging from approximately 1.6 to 2.1-fold. Despite having systematically suppressed DDR gene expressions at baseline, PET-exposed cells mounted a robust and enhanced compensatory transcriptional response to bleomycin-induced damage relative to controls. Notably, the leading gene in the differential, *CDC25A*, was also the most strongly suppressed gene at baseline in PET-exposed cells (Log_2_FC = −1.23, Figure 2b), suggesting that genes chronically downregulated by PET exposure are precisely those whose bleomycin response most strongly diverges between the two groups.

To assess whether functional differences in DNA repair accompanied the differential transcriptional response to bleomycin, residual DNA damage was measured by alkaline comet assay. After 3 h of repair time, PET-exposed cells retained significantly higher levels of residual DNA damage than passage-matched controls (Figure 5c; PET: 12.07 ± 0.85% DNA in tail vs. CN: 2.57 ± 0.29%, mean ± SEM, *p* < 0.001), approximately 4.7-fold difference. This persistent early repair deficit is evident given the elevated transcriptional response and indicates a functional dissociation in which the transcriptional machinery is activated and upregulated, but the capacity to execute repair is compromised.

In PET-exposed cells, despite the broader DDR transcriptional profile observed during challenge, the actual early repair kinetics are impaired relative to controls, indicating that the transcriptional resilience is not functionally equivalent to enhanced repair capacity. This finding is consistent with the baseline suppression of key repair effectors such as *BRCA1* and *RAD51*, required for efficient HR-mediated DSB repair [25,38], which are under expressed in chronically PET-exposed cells. Even if transcriptional levels are maintained during the challenge with bleomycin relative to the suppressed baseline, the absolute abundance of HR proteins in PET-exposed cells during damage induction may be insufficient to mount a repair response equivalent to that of control cells, which began the challenge from a higher baseline expression level. The combination of greater damage induction and impaired early repair in PET-exposed cells, therefore, constitutes a combined vulnerability in which more lesions are created, and fewer are resolved in the critical early window. This early repair deficit is of particular significance given the role of unrepaired DSBs in driving chromosomal instability, translocation events, and aberrant cell cycle progression, processes central to genotoxic carcinogenesis [39,40].

This study provides a model of how chronic, subtoxic nanoplastic exposure compromises DDR function without immediate apparent toxicity. The baseline state of elevated genotoxic damage coupled with transcriptional suppression establishes a cell in a state of reduced genomic vigilance. This state is not acutely lethal, as demonstrated by cells that remain viable at 20 weeks of culture without signs of cytotoxicity, nor is it characterized by clear senescence or rapid apoptosis. Rather, it represents a progressive wearing down of the transcriptional and protein-level infrastructure supporting genomic maintenance. Upon acute challenge with a simple, linear-pathway genotoxin such as MMS or UV-C radiation, this baseline deficit goes largely undetected. However, upon challenge with a complex, multi-component genotoxin requiring coordinated checkpoint activation and assembly of recombination machinery, like bleomycin, the deficit is exposed. The system cannot rapidly synthesize and assemble sufficient functional protein to respond within the critical early window, resulting in cells progressing towards senescence or apoptosis, with accumulating unrepaired DNA lesions.

### 2.5. Environmental Health Relevance of the Chronic Exposure Model

This study shows that nanoplastics, while not explicitly mutagenic in acute exposure scenarios, pose a distinct chronic toxicity risk through their ability to alter DDR capacity. The used model of chronic exposure to PET-NPLs in which subtoxic exposure gradually decreases genomic vigilance, represents a more realistic exposure scenario than those using acute exposure conditions. Real-world human exposure to inhaled nanoplastics occurs not through acute, massive insults, but through persistent, low-level exposure accumulating over a lifetime and the 20-week cell culture model used here provides a more relevant framework than traditional 24 h acute exposure studies.

Importantly, the damage-type-specific sensitization to bleomycin-like lesions (complex DSBs and oxidative lesions) suggests that the real-world health relevance of nanoplastic-induced DDR dysfunction may not manifest immediately, but upon co-exposure to secondary genotoxic stressors. Environmental and occupational agents that generate bleomycin-like damage include ultraviolet A and B radiation (UVA/UVB) [41], ionizing radiation from both natural sources and medical exposures [42], polycyclic aromatic hydrocarbons (PAHs) and other combustion-derived pollutants from vehicular and industrial emissions, diesel exhaust particles and ambient particulate matter (PM_2.5_) [43], and transition metals (including iron, copper, nickel) in air pollution and occupational exposures [44]. In a multi-agent scenario, an individual with a history of chronic nanoplastic exposure could experience disproportionate genotoxic consequences from a DSB-inducing insult compared to a non-exposed individual, substantially increasing their risk of mutagenesis and potentially of cell transformation.

In the broader context of nanoplastic health risk assessment, the damage-type specificity demonstrated here argues for moving beyond generic genotoxicity assays toward a mechanistically oriented evaluation of the DDR consequences of chronic exposure to nanoplastics. The use of three chemically distinct genotoxic agents, rather than a single challenge, is essential for revealing the selectivity of the PET-NPL phenotype, and for connecting it to the specific nature of PET-associated ROS damage. Future studies should extend this approach to include assessment of mutation frequency, micronucleus formation, and chromosomal instability specifically under bleomycin-type or oxidative challenge conditions, to determine whether the functional repair deficit observed here translates into heritable genomic alterations. Additionally, given the strong overlap between the baseline-suppressed gene set and the repair effectors most relevant to bleomycin-induced DSBs, the 20-week PET-NPLs exposure model may provide a useful experimental system for studying how chronic low-level DSB-type genotoxic stress progressively erodes DDR capacity in a respiratory epithelial context relevant to lung cancer risk.

### 2.6. Limitations and Future Research Directions

Limitations of the present study should be acknowledged when interpreting the findings. The exposure concentration (50 µg/mL) is within the range studied in prior acute and subchronic PET-NPL toxicity studies on BEAS-2B cells and is intended to be subtoxic (no gross morphological changes, no major reduction in cell viability across the 20-week period) [10,45]. However, the extent to which this concentration reflects realistic human inhalation exposure to airborne nanoplastics remains uncertain [46,47]. Estimates of environmental nanoplastic exposure vary widely, with uncertainties in both particle size distribution and mass concentration in indoor and outdoor air. Future studies systematically varying concentration and exposure duration and potentially incorporating dosimetry modeling of particle translocation in the respiratory tract, would substantially strengthen the translational relevance of this work and help establish threshold concentrations and exposure durations relevant to human health risk assessment. Regarding the methodology used for the study, the comet assay provides functional corroboration of genotoxic damage and DDR at the cellular level, the present data does not allow direct attribution of the response to any specific molecular pathway. Complementary protein-level analyses, including γH2AX foci as a DSB-specific endpoint, RAD51 foci formation as a readout of HR engagement, and APEX1 or OGG1 enzymatic activity assays, would be required to establish a stronger mechanistic link between the transcriptional changes observed and the functional repair phenotype. Complementary, it should be noted that the genotoxic damage level data reflect population-level averages in the comet assay, and the possibility that the results could be partially contributed to selective loss of the most heavily damaged cells through apoptosis cannot be entirely excluded, despite the absence of obvious cytotoxicity at the concentration and timepoints studied. Future experiments incorporating cell cycle analysis and apoptosis markers at the repair time point should be carried out to address this limitation.

## 3. Materials and Methods

### 3.1. Particle Obtention, Labeling and Characterization

PET-NPLs were obtained from commercially available PET water bottles following a previously established protocol [23,25,48]. Briefly, PET-NPLs were obtained by sanding 12 cm^2^ fragments from the bottom segments of the water bottles using a rotary burr attached to a Dremel 3000 multitool. Sanded PET material (4 g) was passed through a 0.20 mm sieve CISA R-92) and dissolved on pre-heated trifluoroacetic acid, (TFA, 40 mL, 90% *v*/*v*, 60 °C). The initial dissolution was stirred for 2 h until complete dispersion and then continuously agitated at 25 °C for 24 h. An equal volume of TFA 20% *v*/*v* was poured into the mixture and stirred overnight at room temperature. Particles were then centrifuged, resuspended, and dispersed by sonication using an ionic detergent and further washed on Milli-Q water and ethanol. Once dried, particles were resuspended in water and stored at −80 at a concentration of 10 mg/mL. For biological fluorescence applications, a previously reported labeling protocol was used [49,50].

Particle size distribution was calculated by transmission electron microscopy particle count. Working solutions of PET-NPLs were prepared from stock to a concentration of 100 µg/mL, and a single 15 µL drop was deposited on a 5 × 5 mm silicon chip (Ted Pella, Inc., Altadena, CA, USA). The suspension drop was let to dry inside a laminar flow hood, and particles were then visualized using a TEM system Hitachi H-7000 (Hitachi Ltd., Tokyo, Japan). Micrographs were taken from random fields, and the Martin diameter was measured from at least 1000 individual particles. Additionally, dynamic light scattering (DLS) and z-potential measurement were performed on a Zetasizer Ultra and analyzed using the Zetasizer software V7.13 (Malvern Panalytical Ltd., Malvern, UK). Briefly, a DTS 1070 cuvette was filled with a sonicated suspension from the working solution, and the data was acquired at a collection angle of 173°. All measurements were carried out by triplicate. Nanotracking analysis (NTA) was also carried out on a nanoparticle tracking system Nanosight NS300 (Malvern Panalytical Ltd., UK). Briefly, 1 mL of working solution was 1:50 diluted on Milli-Q water, vortexed, transferred to a syringe and injected into the microfluidic system to a final speed of 50 AU and detected using a 488 nm laser using a sCMOS acquisition system (SinceVision, Shenzhen, China). Data was analyzed using NTA software version 3.4 from the same company.

### 3.2. Cell Culture and Long-Term Exposure to PET-NPLs

BEAS-2B cells, a non-tumorigenic bronchial epithelial line transformed with Ad12-SV40 were used in this study and grown in Dulbecco’s modified Eagle’s medium (DMEM, Life Technologies, Hewlett, NY, USA) supplemented with 10% fetal bovine serum (FBS, Biowest, Nuaillé, France) and 2.5 μg/mL of Plasmocin (InvivoGen, San Diego, CA, USA). TrypLE™ Select Enzyme (1X) (Thermo Fisher Scientific, Waltham, MA, USA) was used to detach cells, and the cells were subsequently inactivated in the same DMEM. Cells were maintained in a humidified atmosphere containing 5% CO_2_ at 37 °C. To determine the effect of chronic exposure to PET-NPLs in the cell model, 2 × 10^5^ BEAS-2B cells were seeded in triplicate in 75 cm^2^ flasks in 10 mL of medium and continuously exposed to 50 µg/mL of PET-NPLs for 20 weeks by doing weekly cell passages at around 80% confluency and replacing the cell culture medium twice a week with fresh DMEM with PET-NPLs. To ensure any effects observed after 20 weeks were a product of the exposure to the NPLs and not continuous cell passage, non-exposed passage-matched cells were seeded in parallel in triplicate under the same culture conditions for 20 weeks.

### 3.3. Particle Internalization Using Confocal Microscopy

Internalization of labelled PET-NPLs was determined by confocal microscopy to confirm the uptake of these particles in BEAS-2B cells after 20 weeks of continuous exposure. A total of 1 × 10^4^ cells/well of chronically exposed cells were seeded in an 8-well µ-Slide chambered coverslip (Ibidi GmbH, Grafelfing, Germany) and treated with 50 µg/mL of labelled PET-NPLs for 24 h. Cells were then washed twice with warm growth medium and stained using nuclei marker Hoechst 33342 (ex: 405 nm, em: 415–503 nm) and cell membrane marker Cellmask (ex: 633 nm, em: 645–786 nm). Leica TCS CP5 confocal microscope was used to visualize the cells and confirm particle internalization against non-treated passage-matched controls.

### 3.4. DNA Damage Induction

DNA damage response (DDR) by the base excision repair (BER), nucleotide excision repair (NER), and homologous recombination (HR) pathway was evaluated after DNA damage induced by the alkylating agent methyl methanesulfonate (MMS) (Sigma-Aldrich, Co., St. Louis, MO, USA), ultraviolet-C (UV-C) radiation, and bleomycin (Sigma-Aldrich, Co., St. Louis, MO, USA), respectively. PET-exposed BEAS-2B cells were seeded in duplicate for each condition in 12-well plates at 2 × 10^5^ cells/well and, after 24 h of growth in DMEM with 50 μg/mL of PET-NPLs, treated with 1 μM MMS for 30 min, 10 mJ/cm^2^ of UV-C, or 25 μg/mL of bleomycin for 1 h. The treatment doses were selected so they would induce significant DNA damage to initiate DDR without showing cell toxicity. Cells were washed three times with PBS 1X and allowed to be repaired in DMEM containing PET-NPLs for 0 and 3 h. DDR was also evaluated in passage-matched control cells after damage induction. DNA genotoxic damage was evaluated using the comet assay described in the following section. Three different experiments (n = 3) were carried out for each condition.

### 3.5. The Comet Assay

Genotoxic DNA damage induced by MMS, UV-C radiation, and bleomycin immediately after treatment and after 3 h of repair time was evaluated using the alkaline comet assay, as described [51]. Before damage induction and following 0 and 3h of repair time after induction of DNA damage, BEAS-2B cells were washed with PBS 1X, detached, collected, and centrifuged (300 rcf at 4 °C, 8 min). The supernatant was removed, and the cell pellet was resuspended in cold 1X PBS to a cell density of 1 × 10^6^ cells/mL. The cell suspension was diluted (1:10) in 0.75% low melting point agarose (Invitrogen, Carlsbad, CA, USA) at 37 °C, and the mix was placed on Gelbond^®^ films (GBF, Life Sciences, Vilnius, Lithuania) as 7 μL drops. After the agarose was solidified, the gels were placed in lysis buffer (0.01 M Tris Base, 0.2 M NaOH, 2.5 M NaCl, 0.1 M EDTA, 1% Triton X-100, 1% N-lauroyl sarcosine, 10% DMSO; pH 10) at 4 °C for 2 h. GBFs were washed twice with cold enzyme buffer (0.5 mM EDTA, 0.04 M HEPES, 0.1 M KCl, 0.2 mg/mL; pH 8), and incubated in the same buffer for 50 min at 4 °C. A second incubation in this buffer was carried out for 30 min at 37 °C. GFBs were washed with electrophoresis buffer (1 mM EDTA, 0.3 M NaOH; pH 13.4) and then incubated in the same buffer for 25 min to allow DNA unwinding before electrophoresis (300 mA, 20 V, 20 min). Two washes with 1X cold PBS were performed before GFBs were fixed in absolute ethanol and allowed to dry overnight. Staining was carried out with SYBR Gold in TE buffer (10 mM Tris Base, 1 mM EDTA; pH 8) (1:2500), and comets were scored at a 20X magnification with an Olympus BCXC50 microscope (Olympus Optical CO., Hamburg, Germany) using the Komet 5.5 Image analysis system (Kinetic Imaging Ltd., Liverpool, UK). DNA percentage in the comet’s tail was recorded as a measurement of genotoxic damage, and data were normalized to the % DNA in the tail of cells before DNA damage induction. GraphPad Prism Software 8.0.2 (GraphPad, San Diego, CA, USA) was used to analyze the data using the Mann–Whitney U test (n = 3) test with a 95% confidence interval.

### 3.6. RNA Extraction After DNA Damage Induction

PET-exposed and passage-matched BEAS-2B cells were seeded in 75 cm^2^ flasks at 2 × 10^6^ cells/flask in triplicate (n = 3). DNA damage was induced with bleomycin as described in Section 2.4, and controls without induced damage were used to determine baseline expression. Following instructions from the manufacturer, cells were detached and centrifuged for 8 min at 200 rcf to obtain a cell pellet for RNA extraction using the RNeasy^®^ Mini Kit following the on-column DNase digestion step (Qiagen, Hilden, Germany). RNA concentrations were determined using a Nanodrop Spectrophotometer (Thermo Fisher Scientific Technologies, Waltham, MA, USA) to confirm at least 40 μg/mL of RNA per sample.

### 3.7. Gene Expression Using the RT^2^ Profiler PCR Array Human DNA Damage Signaling Pathway

Baseline RNA expression and after induced damage with bleomycin were determined following the protocol for RT^2^ Profiler™ PCR Array Human DNA Damage Signaling Pathway (PAHS-029ZG-4, QIAGEN, Germantown, MD, USA). RT^2^ First Strand Kit was used for cDNA synthesis, starting with 400 ng/sample of RNA extracted in the previous step (Section 2.6) and following the manufacturer’s instructions for Real-Time PCR Array Format G (4 × 96). The array profiles 84 DNA damage response (DDR) genes across a 4 × 96-well plate format, with the remaining wells occupied by five housekeeping genes (*ACTB*, *B2M*, *GAPDH*, and *RPLP0*) and proprietary genomic DNA contamination, reverse transcription efficiency, and PCR efficiency control elements. LightCycler^®^ 480 (Roche Diagnostics Ltd., Basel, Switzerland) was used for the real-time PCR (1 cycle of 10 min at 95 °C, 45 cycles of 15 s at 95 °C followed by 1 min at 60 °C) and Cp data was obtained with the LightCycler^®^ 480 SW 1.5.1.62 Software (Roche Diagnostics Ltd., Rotkreuz, Switzerland).

### 3.8. Data Analysis of the RT^2^ Profiler PCR Array Human DNA Damage Signaling Pathway

Gene expression data from the RT^2^ Profiler™ PCR Array Human DNA Damage Signaling Pathway were analyzed manually using the ΔΔCt method. For each gene of interest and each replicate, the normalized expression value (ΔCt) was calculated as the difference between the raw Cp of the gene of interest and the arithmetic mean Cp of the four reference genes within the same replicate (ΔCt = Cp_gene of interest_ − mean Cp_ACTB, B2M, GAPDH, RPLP0_). ΔCt values were calculated independently for each replicate before any averaging, ensuring that inter-replicate variability was fully propagated through the downstream calculations. Gene expression comparisons were performed between PET-NPL-exposed (PET) and passage-matched control (CN) BEAS-2B cells at baseline without genotoxic challenge, and following bleomycin treatment. For the baseline comparison, the ΔΔCt for each replicate was calculated as ΔΔCt_baseline_ = ΔCt(PET_untreated_) − ΔCt(CN_untreated_). For the bleomycin comparison, the ΔΔCt was designed to capture the differential transcriptional response to genotoxic challenge in PET-exposed relative to control cells, above and beyond the shared genotoxin-induced response common to both groups. This was calculated per replicate as ΔΔCt_Bleomycin_ = [ΔCt(PET_Bleomycin_) − ΔCt(PET_Basal_)] − [ΔCt(CN_Bleomycin_) − ΔCt(CN_Basal_)]. The fold change (FC) was computed as FC = 2^Log^_2_^FC^. A biological relevance threshold of |Log_2_FC| > Log_2_(1.5) ≈ 0.585 (corresponding to a minimum ≥1.5-fold difference in expression) was applied as the primary significance criterion, consistent with standard practice in pathway-focused RT^2^ Profiler PCR Array analyses. Genes meeting this threshold are referred to throughout as showing a biologically relevant change.

## 4. Conclusions

The present study demonstrates that chronic (20-week) exposure to polyethylene terephthalate nanoplastics (PET-NPLs) at 50 µg/mL induces a state of genomic instability in BEAS-2B human bronchial epithelial cells, characterized by a significant increase in basal DNA damage and a systematic, cross-pathway suppression of the DNA damage response (DDR) transcriptome under steady-state conditions. The results showed that this chronic exposure does not result in a total collapse of repair capacity but rather a lesion-type-specific modulation of the DDR. While the exposed cells had a similar sensitivity to the passage-matched controls under alkylation damage by MMS and pyrimidine dimers by UV-C radiation, they exhibited a significant sensitization and functional bottleneck when challenged with the complex strand breaks induced by bleomycin, revealing a vulnerable ATM/ATR-dependent homologous recombination pathway. Evaluation of the transcriptomic profile of the exposed cells upon challenge with bleomycin showed a transition from a suppressed baseline into a state in which the cell recognizes damage and initiates transcriptional responses but fails to execute effective repair as demonstrated by the significantly higher damage retained in comparison with the control cells after 3 h of repair time. The findings highlight the need for nanoplastic risk assessments to transition from acute toxicity paradigms toward conditions that better represent those of real-life exposure, including chronic low-doses and the possibility of co-exposure with other stressors, and mechanistically resolved evaluations of the DDR to accurately capture the long-term carcinogenic risks posed by persistent environmental pollutants.

## Data Availability

The original contributions presented in this study are included in the article/Supplementary Material. Further inquiries can be directed to the corresponding authors.

## Supplementary Materials

The following supporting information can be downloaded at: https://www.mdpi.com/article/10.3390/ijms27115031/s1.

## Abbreviations

BEAS-2B	Human bronchial epithelial cell line
BER	Base excision repair
CN	Control cells
DDR	DNA damage repair
DLS	Dynamic Light Scattering
DSB	Double-strand breaks
HR	Homologous recombination
MMS	Methyl methanesulfonate
MNPLs	Microplastics
NER	Nucleotide excision repair
NPLs	Nanoplastics
NTA	Nanoparticle tracking analysis
PAHs	Polycyclic aromatic hydrocarbons
PET	Polyethylene terephthalate
PET-NPLs	PET Nanoplastics
PM2.5	Ambient particulate matter
ROS	Reactive oxygen species
TEM	Transmission electron microscopy
UVA	Ultraviolet radiation A
UVB	Ultraviolet radiation B
UV-C	Ultraviolet radiation C
