# Supplementary material for: Long-Term PET-Nanoplastic Exposure Alters DNA Damage Response Capacity in BEAS-2B Human Bronchial Epithelial Cells
Source: Int J Mol Sci. 2026 Jun 2;27(11):5031. doi: 10.3390/ijms27115031 (PMC13256201; doi:10.3390/ijms27115031)
Supplement: Supplementary file 1 [file ijms-27-05031-s001.zip › ijms-4309626-supplementary.pdf]

Long-Term PET-Nanoplastic Exposure Alters DNA Damage Response Capacity in BEAS-2B Human Bronchial Epithelial Cells

Michelle Morataya-Reyes[1], Aliro Villacorta[1,2], Raquel Egea[1], Juan Martín-Pérez[1], Javier Gutiérrez-García[1], Susana Pastor[1], Ricard Marcos[1,§], Alba Hernández[1,§]

SUPPLEMENTARY MATERIAL

Table S1. ΔCt values of the 84 genes included in the RT² Profiler™ PCR Array Human DNA Damage Signaling Pathway. The columns marked in green present the mean values of 3 replicates of the change in basal expression between PET-exposed BEAS-2B cells (20 weeks) and the passage-matched controls, including the mean Log_2FC and the FC. The column in blue presents the change in expression for the interaction of PET-exposure and Bleomycin challenge (ΔΔCt) with the respective mean Log_2FC and FC values. (ΔΔCt = mean of replicate ΔΔCt values. $Log_2FC = -\Delta\Delta Ct$. $FC = 2^{Log_2FC}$. Within-group: ΔΔCt = ΔCt(PET) − ΔCt(CN). Between-group: ΔΔCt = [ΔCt(PET,Bleomycin−ΔCt(PET,0)] − [ΔCt(CN,Bleomycin)−ΔCt(CN,0)]. Yellow = $|Log_2FC| > 0.585$ (≥1.5×), blue bold letters → downregulated, red bold letters → upregulated).

Gene / Well		CN → PET			ΔΔCt | BLEO (PET vs CN)		
Gene	Well	Mean ΔΔCt	Mean Log_2FC	FC	Mean ΔΔCt	Mean Log_2FC	FC
ABL1	A01	**1.1180**	**-1.12**	**0.46**	**-1.0733**	**1.0733**	**2.10**
APEX1	A02	**0.7180**	**-0.72**	**0.61**	**-0.7633**	**0.7633**	**1.70**
ATM	A03	**0.6780**	**-0.68**	**0.63**	-0.5267	0.5267	1.44
ATR	A04	0.5113	-0.5113	0.7016	-0.3433	0.3433	1.27
ATRIP	A05	**0.7747**	**-0.77**	**0.58**	**-0.9400**	**0.9400**	**1.92**
ATRX	A06	**0.8413**	**-0.84**	**0.56**	-0.4833	0.4833	1.40
BARD1	A07	**0.5947**	**-0.59**	**0.66**	**-0.7133**	**0.7133**	**1.64**
BAX	A08	0.5747	-0.5747	0.6714	**-0.6933**	**0.6933**	**1.62**
BBC3	A09	**0.7080**	**-0.71**	**0.61**	**-0.8833**	**0.8833**	**1.84**
BLM	A10	0.5347	-0.5347	0.6903	-0.2967	0.2967	1.23
BRCA1	A11	**0.6913**	**-0.69**	**0.62**	**-0.6700**	**0.6700**	**1.59**
BRIP1	A12	**0.6113**	**-0.61**	**0.65**	-0.2700	0.2700	1.21
CDC25A	B01	**1.2280**	**-1.23**	**0.43**	**-1.0900**	**1.0900**	**2.13**
CDC25C	B02	0.5147	-0.5147	0.7000	-0.5633	0.5633	1.48
CDK7	B03	0.5247	-0.5247	0.6951	-0.4867	0.4867	1.40
CDKN1A	B04	0.5113	-0.5113	0.7016	-0.5367	0.5367	1.45
CHEK1	B05	**0.6113**	**-0.61**	**0.65**	-0.4667	0.4667	1.38
CHEK2	B06	0.5047	-0.5047	0.7048	-0.4167	0.4167	1.33
CIB1	B07	**0.6013**	**-0.60**	**0.66**	**-0.5867**	**0.5867**	**1.50**
CRY1	B08	0.5847	-0.5847	0.6668	-0.4967	0.4967	1.41
CSNK2A2	B09	0.4080	-0.4080	0.7537	-0.4667	0.4667	1.38
DDB1	B10	0.5513	-0.5513	0.6824	-0.5400	0.5400	1.45
DDB2	B11	0.5580	-0.5580	0.6792	**-0.7233**	**0.7233**	**1.65**
DDIT3	B12	**0.7680**	**-0.77**	**0.59**	**-0.6433**	**0.6433**	**1.56**
ERCC1	C01	**0.7147**	**-0.71**	**0.61**	-0.5600	0.5600	1.47
ERCC2	C02	**0.6447**	**-0.64**	**0.64**	-0.5667	0.5667	1.48
EXO1	C03	0.5047	-0.5047	0.7048	-0.3167	0.3167	1.25
FANCA	C04	0.4747	-0.4747	0.7196	-0.5333	0.5333	1.45
FANCD2	C05	0.4980	-0.4980	0.7081	-0.3833	0.3833	1.30
FANCG	C06	0.5013	-0.5013	0.7065	**-0.7100**	**0.7100**	**1.64**
FEN1	C07	0.4747	-0.4747	0.7196	-0.4667	0.4667	1.38
GADD45A	C08	**0.6813**	**-0.68**	**0.62**	-0.2100	0.2100	1.16

GADD45G	C09	**0.9347**	**-0.93**	**0.52**	-0.3267	0.3267	1.25
H2AFX	C10	**0.5947**	**-0.59**	**0.66**	-0.5167	0.5167	1.43
HUS1	C11	0.5547	-0.5547	0.6808	-0.4333	0.4333	1.35
LIG1	C12	**0.6647**	**-0.66**	**0.63**	**-0.6133**	**0.6133**	**1.53**
MAPK12	D01	0.5513	-0.5513	0.6824	-0.4800	0.4800	1.39
MBD4	D02	0.4380	-0.4380	0.7382	-0.5500	0.5500	1.46
MCPH1	D03	**0.6180**	**-0.62**	**0.65**	-0.4433	0.4433	1.36
MDC1	D04	**0.5913**	**-0.59**	**0.66**	-0.3800	0.3800	1.30
MLH1	D05	**0.6080**	**-0.61**	**0.66**	-0.5167	0.5167	1.43
MLH3	D06	0.2180	-0.2180	0.8598	-0.2200	0.2200	1.16
MPG	D07	0.5280	-0.5280	0.6935	**-0.6367**	**0.6367**	**1.55**
MRE11A	D08	0.4513	-0.4513	0.7314	-0.3333	0.3333	1.26
MSH2	D09	0.5647	-0.5647	0.6761	-0.4100	0.4100	1.33
MSH3	D10	0.4547	-0.4547	0.7297	-0.3500	0.3500	1.27
NBN	D11	0.4513	-0.4513	0.7314	-0.2367	0.2367	1.18
NTHL1	D12	0.3747	-0.3747	0.7713	-0.4700	0.4700	1.39
OGG1	E01	**0.6913**	**-0.69**	**0.62**	**-0.7000**	**0.7000**	**1.62**
PARP1	E02	0.4580	-0.4580	0.7280	-0.4933	0.4933	1.41
PCNA	E03	0.3613	-0.3613	0.7784	-0.3867	0.3867	1.31
PMS1	E04	0.4847	-0.4847	0.7147	-0.1933	0.1933	1.14
PNKP	E06	0.5247	-0.5247	0.6951	-0.4533	0.4533	1.37
PPM1D	E07	0.5613	-0.5613	0.6777	-0.5400	0.5400	1.45
PPP1R15A	E08	0.5180	-0.5180	0.6983	-0.4200	0.4200	1.34
PRKDC	E09	**0.6080**	**-0.61**	**0.66**	-0.2033	0.2033	1.15
RAD1	E10	0.5047	-0.5047	0.7048	-0.3967	0.3967	1.32
RAD17	E11	**0.6447**	**-0.64**	**0.64**	-0.3700	0.3700	1.29
RAD18	E12	0.3747	-0.3747	0.7713	-0.2767	0.2767	1.21
RAD21	F01	**0.8147**	**-0.81**	**0.57**	**-0.7367**	**0.7367**	**1.67**
RAD50	F02	0.4147	-0.4147	0.7502	-0.4100	0.4100	1.33
RAD51	F03	0.5380	-0.5380	0.6887	**-0.6200**	**0.6200**	**1.54**
RAD51B	F04	0.4380	-0.4380	0.7382	-0.3100	0.3100	1.24
RAD9A	F05	**0.6047**	**-0.60**	**0.66**	**-0.5867**	**0.5867**	**1.50**
RBBP8	F06	0.3347	-0.3347	0.7930	-0.3567	0.3567	1.28
REV1	F07	0.4447	-0.4447	0.7348	-0.2667	0.2667	1.20
RNF168	F08	0.4980	-0.4980	0.7081	-0.4667	0.4667	1.38
RNF8	F09	0.2080	-0.2080	0.8657	-0.2433	0.2433	1.18
RPA1	F10	0.5647	-0.5647	0.6761	-0.5767	0.5767	1.49
SIRT1	F11	0.4180	-0.4180	0.7485	-0.4167	0.4167	1.33
SMC1A	F12	0.4113	-0.4113	0.7519	-0.3300	0.3300	1.26
SUMO1	G01	0.2713	-0.2713	0.8286	0.0533	-0.0533	0.96
TOPBP1	G02	0.2180	-0.2180	0.8598	-0.2233	0.2233	1.17
TP53	G03	0.3213	-0.3213	0.8003	-0.3767	0.3767	1.30
TP53BP1	G04	0.2880	-0.2880	0.8190	-0.0800	0.0800	1.06
TP73	G05	-0.2953	0.2953	1.2272	-0.3367	0.3367	1.26
UNG	G06	0.0847	-0.0847	0.9430	-0.0967	0.0967	1.07
XPA	G07	0.0313	-0.0313	0.9785	-0.0533	0.0533	1.04

XPC	G08	0.1947	-0.1947	0.8738	-0.1133	0.1133	1.08
XRCC1	G09	0.2947	-0.2947	0.8153	-0.1933	0.1933	1.14
XRCC2	G10	0.3080	-0.3080	0.8078	-0.0300	0.0300	1.02
XRCC3	G11	0.2213	-0.2213	0.8578	-0.3667	0.3667	1.29
XRCC6	G12	0.2747	-0.2747	0.8266	-0.0733	0.0733	1.05

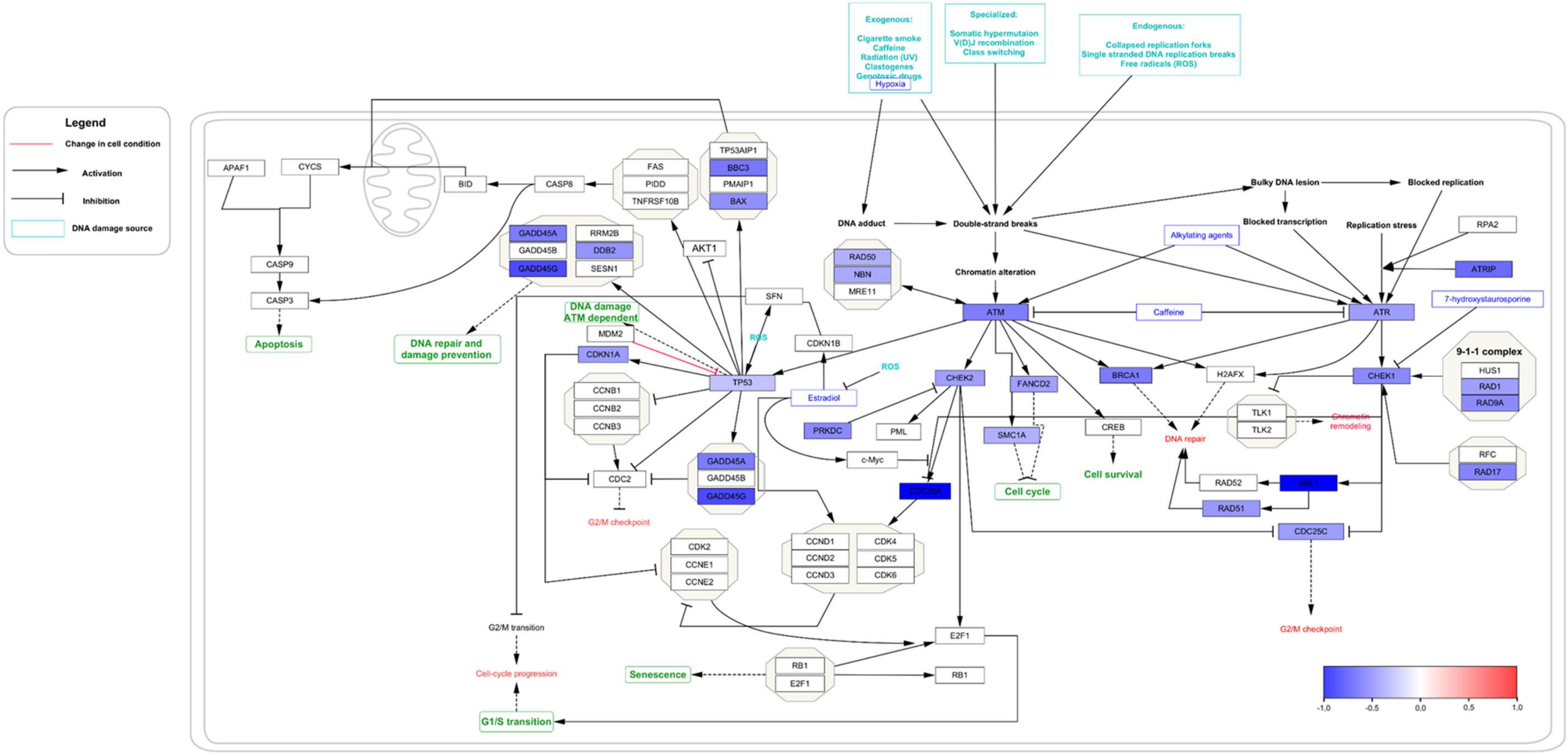

Figure S1. Mapping of baseline DDR gene expression changes in PET-NPL-exposed BEAS-2B cells onto the DNA Damage Response Pathway (WikiPathways WP707). Gene expression was compared between passage-matched control (CN) and PET-NPL-exposed (PET) BEAS-2B cells after 20 weeks of continuous exposure in the absence of genotoxic challenge, using the RT² Profiler™ PCR Array Human DNA Damage Signaling Pathway. The WP707 pathway is organized around *ATM* and *ATR* as central damage-sensing kinases, with branches connecting upstream damage sources to downstream checkpoint, repair, and apoptotic effectors. The p53-dependent branch of the DDR governs cell cycle arrest, DNA repair coordination, and the apoptotic response to irreparable damage, and several of its canonical transcriptional effectors were among the

most consistently attenuated genes in PET-NPL-exposed cells: *GADD45A* and *GADD45G*, which mediate G2/M checkpoint arrest and promote NER; *BBC3*/PUMA and *DDIT3*, pro-apoptotic effectors that execute p53-dependent cell death in response to irreparable damage; and *CDC25A*, whose chronic suppression indicates attenuated coordination between checkpoint signaling and cell cycle re-entry. Gene nodes within the network are colored according to the Log_2FC of PET-exposed relative to control cells: blue indicates downregulation in PET-exposed cells, with color intensity proportional to the magnitude of suppression ($Log_2FC \leq -0.585$); red indicates upregulation ($Log_2FC \geq +0.585$); nodes with no detectable change ($|Log_2FC| < 0.585$) are shown in white.
